# Three axonal projection routes of individual pyramidal cells in the ventral CA1 hippocampus

**DOI:** 10.3389/fnana.2014.00053

**Published:** 2014-06-25

**Authors:** Antónia Arszovszki, Zsolt Borhegyi, Thomas Klausberger

**Affiliations:** ^1^Department of Cognitive Neurobiology, Center for Brain Research, Medical University of ViennaVienna, Austria; ^2^MRC Anatomical Neuropharmacology Unit, Department of Neuropharmacology, Oxford UniversityOxford, UK

**Keywords:** ventral CA1, pyramidal neuron, axonal projection, apical dendrite, calbindin, norbin

## Abstract

Pyramidal cells of the ventral hippocampal CA1 area have numerous and diverse distant projections to other brain regions including the temporal and parietal association areas, visual, auditory, olfactory, somatosensory, gustatory, and visceral areas, and inputs to the amygdalar and prefrontal-orbital-agranular insular region. In addition, their differential expression of proteins like calbindin provides further indications for cellular diversity. This raises the possibility that the pyramidal cells may form subpopulations participating in different brain circuitries. To address this hypothesis we applied the juxtacellular labeling technique to fill individual pyramidal cells in the ventral hippocampus with neurobiotin in urethane anesthetized rats. For each labeled pyramidal cell we determined soma location, dendritic arborizations and selective expression of calbindin and norbin. Reconstruction and mapping of long-range axonal projections were made with the Neurolucida system. We found three major routes of ventral CA1 pyramidal cell projections. The classical pathway run caudo-ventrally across and innervating the subiculum, further to the parahippocampal regions and then to the deep and superficial layers of entorhinal cortex. The other two pathways avoided subiculum by branching from the main axon close to the soma and either traveled antero- and caudo-ventrally to amygdaloid complex, amygdalopiriform-transition area and parahippocampal regions or run antero-dorsally through the fimbria-fornix to the septum, hypothalamus, ventral striatum and olfactory regions. We found that most pyramidal cells investigated used all three major routes to send projecting axons to other brain areas. Our results suggest that the information flow through the ventral hippocampus is distributed by wide axonal projections from the CA1 area.

## Introduction

The hippocampus, a C-shape cortical structure, is a part of the limbic system. The hippocampus can be divided along the longitudinal (or septotemporal) axis into dorsal (septal pole), intermediate and ventral (temporal pole) parts. Many studies have focused on the dorsal part, but extrapolations of observations to more temporal parts seem difficult because anatomical (Amaral and Witter, 1989; Amaral and Lavenex, 2007), electrophysiological (Jung et al., 1994; Kjelstrup et al., 2008), and systemic (Moser and Moser, 1998) investigations revealed differences along the septotemporal (dorsal–ventral) hippocampal axis.

The dorsal hippocampus (DH) is associated with spatial navigation and episodic memory as well as associative learning (O'keefe, 1976; Bliss and Collingridge, 1993; Eichenbaum, 1999; Squire, 2007). The ventral hippocampus (VH) is involved in modulating stress responses and motivated or emotional behaviors furthermore it may participate in variety of autonomic, neuroendocrine, defensive, ingestion-related and reproductive control systems (Moser and Moser, 1998; Bannerman et al., 2004; Kjelstrup et al., 2008). This functional difference is also supported by lesion studies, DH lesions cause impaired spatial learning (Moser et al., 1995) while VH lesions affect non-spatial functions like altered anxiety in rats (Bannerman et al., 2002). This dissociation seems to be matched also by a difference in synaptic connectivity. Anatomical investigations pointed out that the input and output connections of the dorsal and ventral hippocampus are different (Swanson and Cowan, 1977). The DH receives input from the dorsal entorhinal cortex (Dolorfo and Amaral, 1998) carrying visual-spatial information, on the other hand VH is innervated more heavily by the amygdala (Risold and Swanson, 1996) and thus may participate more in the processing of emotional information. Differences between the dorsal and ventral part have been recognized also by the observation that several distinct genetic markers are differentially expressed along the septo-temporal axis of the hippocampus (Dong et al., 2009).

The pyramidal neurons of the CA1 area represent the main output of the hippocampus. They heavily project to the subiculum and innervate the deeper layers of entorhinal cortex (Amaral and Witter, 1989). In addition, pyramidal cells in the ventral CA1 area also send long projections to many other brain areas including amygdalar complex, hypothalamus, prefrontal cortex, nucleus accumbens, olfactory regions, auditory cortex, visual cortex (Andersen et al., 1971; Swanson and Cowan, 1977; Van Groen and Wyss, 1990; Cenquizca and Swanson, 2006, 2007). Pyramidal neurons were suggested to participate in two separate circuits in the VH (Canteras and Swanson, 1992; Risold and Swanson, 1996), one controlling the neuroendocrine activities, connecting the most ventral tip of VCA1 and VSub, with the posterior part of amygdalar nucleus (BLP), the ventral part of the lateral septum (LSV) and the antero-medial part of bed nucleus stria terminalis (BST). The second circuitry may play role in fear learning via direct connections from VH to the central amygdaloid nucleus capsular part (CeC) (Cenquizca and Swanson, 2007).

Most data describing these projections from the hippocampus were achieved by bulk labeling studies, in which the axons of a population of pyramidal neurons were visualized with retrograde and/or anterograde tracers (Van Groen and Wyss, 1990; Canteras and Swanson, 1992; Cenquizca and Swanson, 2006, 2007) covering the possible differences among pyramidal neurons. These techniques provided important knowledge on the projection areas of the ventral CA1 hippocampus, but have limitations in distinguishing the projections from individual cells (Ishikawa and Nakamura, 2006). However, CA1 pyramidal neurons may not represent a homogenous cell type. Distinct types of neuron can be differentiated based on their axonal arborization, subcellular synaptic targets, size and shape of soma, dendritic specializations or based on their molecular expression profile or based on their membrane properties and firing in relation to different EEG activities. There has been growing evidence that a diversity of pyramidal neurons exist in the CA1 area. Diverse firing patterns and dendritic arborizations have been observed for CA1 pyramidal neurons (Harris et al., 2001; Spruston, 2008; Mizuseki et al., 2011). Based on immunolabeling for the Ca^2+^-binding protein calbindin (CB), immunopositive cells are found in the compact layer of the str. pyramidale whereas immunonegative cells are preferentially found in the part of the pyramidal layer that is closer to the str. oriens (Baimbridge and Miller, 1982). The calcium-binding protein may play a role in synaptic plasticity, as reduced amount of CB in transgenic mice caused impaired spatial learning, deficits in memory and changes in LTP (Molinari et al., 1996). Furthermore, CB+ cells close to stratum radiatum develops later than the CB- neurons (Baimbridge et al., 1991). There are more place cells in the deep layer, where CB− cells localized, while the more superficial (mostly CB+) neurons seems to play a role in non-spatial information flow in DH (Mizuseki et al., 2011).

In addition, large strongly immunopositive cells have been observed, mostly, but not exclusively in deep str. pyramidale (Klausberger and Somogyi, 2008). Other pyramidal cells have been observed in str. radiatum (Maccaferri and McBain, 1996; Gulyas et al., 1998; Bullis et al., 2007) and in str. pyramidale, which are immunonegative for calbindin and project to the accessory olfactory bulb (Van Groen and Wyss, 1990).

In addition to CB, norbin (neurochondrin) might be another protein that is differentially expressed in CA1 pyramidal cells. Norbin is a neurite outgrowth-related protein which is expressed in developmental and adult brain in mice (Istvanffy et al., 2004) and rats (Shinozaki et al., 1999). Its functional connection with plasticity via CaMKII (Dateki et al., 2005) and mGluR5 (Wang et al., 2009) was shown. This protein is highly expressed in many brain regions including the hippocampus, mainly in pyramidal neurons and granule cells (Istvanffy et al., 2004).

In order to test whether individual pyramidal cells have specific axonal projections, dendritic branching and molecular expression profiles, we performed juxtacellular labeling of individual pyramidal cells in the ventral CA1 hippocampus to visualize axonal and dendritic arborizations and to test the molecular expression profiles.

## Materials and methods

### Juxtacellular labeling

Experimental procedures were performed on adult male Sprague Dawley rats weighing 290–400 g, and were performed in accordance with licenses approved by the Austrian Ministry of Science. Anesthesia was induced with 4% isoflurane in oxygen, followed by urethane injection (1.25 g/kg body weight, i.p.) and for long-term anesthesia additional doses of ketamine/xylazine mixture (20 and 2 mg/kg, respectively) was injected, as needed. Body temperature was maintained at 37°C with a heating pad and electrocardiographic activity was constantly monitored during the entire experiment (up to 24 h from the first urethane injection). During the surgery the head was fixed in a stereotaxic frame and all wound margins were treated with the local anesthetic Xylocaine. For the juxtacellular labeling we were using a glass electrode (12–24 MΩ) filled with 1.5 or 3% Neurobiotin (Vector Laboratories) in 0.5 M NaCl. Coordinates relative to bregma with a 10° angle mediolateral were as follows: anteroposterior, −4.6–5.4 mm; mediolateral, 4; dorsoventral, 7–8.5 mm. Secondary electrodes were also placed to the dorsal CA1 stratum pyramidale. After the juxtacellular labeling rats were taken from the frame to a box and anesthesia was maintained with additional doses of urethane and ketamine/xylazine mixture as required.

### Tissue processing, anatomical analysis, cell reconstruction, and mapping

Thirteen to twenty hours after juxtacellular labeling rats were perfused with saline followed by 20 min. fixation with 4% paraformaldehyde (PFA), 15% (v/v) saturated picric acid, and 0.05% glutaraldehyde (GA) in 0.1 M phosphate buffer. Serial coronal sections, horizontal section in one case (A23) or sagittal sections in 2 cases (A71/A73) were cut (thickness: 70 μm) from the entire right hemisphere with a vibratome (Leica VT 1000S). The labeled cells were visualized by streptavidin-conjugated Alexa 488 (1:1000; Invitrogen) or streptavidin-conjugated AMCA (1:100; Jackson Immunoresearch Laboratories).

Immunohistochemistry was perfomed to test the expression profile of pyramidal neurons with primary antibodies immunoreactive for calbindin (CB) raised in rabbit (1:5000, Swant CB-D28k) and norbin (N) raised in rabbit (1:100, Fab Gennix NORB/Neurochondrin 101AP), which were detected, by Cy3 (1:400; Jackson Immunoresearch Laboratories) or A488 (1:1000; Jackson Immunoresearch Laboratories) -conjugated secondary antibodies. The CB antibody was characterized previously (Celio et al., 1990; Airaksinen et al., 1997). Pictures from the dendrites were taken from the surface of sections. The specificity of the norbin antibody was tested by the company with western blot analysis. For the immunohistochemical analysis performed here we observed a labeling pattern of this antibody similar to that described in a previous study using a different antibody against norbin (Shinozaki et al., 1999). Norbin is a cytosolic protein localized in the somatodendritic region of principal neurons and some interneurons in the hippocampus, cerebellum and cerebral cortex. We were using control sections (without surgery) in each immunoreaction of labeled cells and additional control experiments were carried out without primary antibody. Immunohistochemical analysis was performed on an epifluorescence (Olympus BX61) or confocal (Leica TCS SP5) microscope.

After immunohistochemistry, sections were demounted and were incubated in ABC solution (elite; 1:100; Vector Laboratories) and then converted by the glucose oxidase method (Losonczy et al., 2002) either with or without Ni^2+^ intensification using DAB as chromogene followed by 1% osmium tetroxide to enhance contrast. After dehydration, sections were embedded in epoxy resin (Durcupan, Fluka). The sections that were not processed for fluorescence evaluation were freeze-thawed to enhance penetration before ABC and during the dehydration 1% uranyl-acetate was applied to increase contrast for possible electron microscopic examination.

The borders between the subareas of the hippocampus like CA1, CA2, CA3, subiculum are difficult to define in the ventral hippocampus. To identify CA2 area, α-actinin immunostaining were carried out (Ratzliff and Soltesz, 2001) that labeled pyramidal neurons selectively in this region. CB immunolabeling was also used to outline VCA1 and VSub. The transition between the two structures can be recognized as the CB positive pyramidal somata become sparse and more widespread, and the CB labeled apical dendrites of the pyramidal cells indicate CA1 radiatum and lacunosum-moleculare distinct to the one homogenous molecular layer of the subiculum. These two immunoreactions were applied to serial sections of the entire hippocampus resulting in a reference map that, together with brain atlases, served to define the positions of the cell somata.

The somatodendritic and axonal arborizations of labeled cells were examined under a light microscope. All neurobiotin filled axonal and dendritic arborizations were followed throughout the brain and parts were reconstructed using the Neurolucida system (version 10. MicroBrightField) with a 100× oil-immersion objective (Olympus UPlanFLN, NA 1.30). Reconstructed sections were correlated with two rat brain atlases (Swanson, 2004; Paxinos and Watson, 2007).

Beside the widely used coronal sectioning, we applied horizontal (A23) and parasagittal (A71, A73) cutting as well to gain easier handling of the large number of sections and improve reconstruction. However, the resolution of the atlases (Swanson, 2004; Paxinos and Watson, 2007) was not as precise in these alternative planes as it turned out to be necessary for accurate localization of the axons and therefore most brains were cut in the traditional coronal plane.

## Results

### Juxtacellular labeling of pyramidal cells in the ventral CA1 (VCA1)

**Figure 1:**
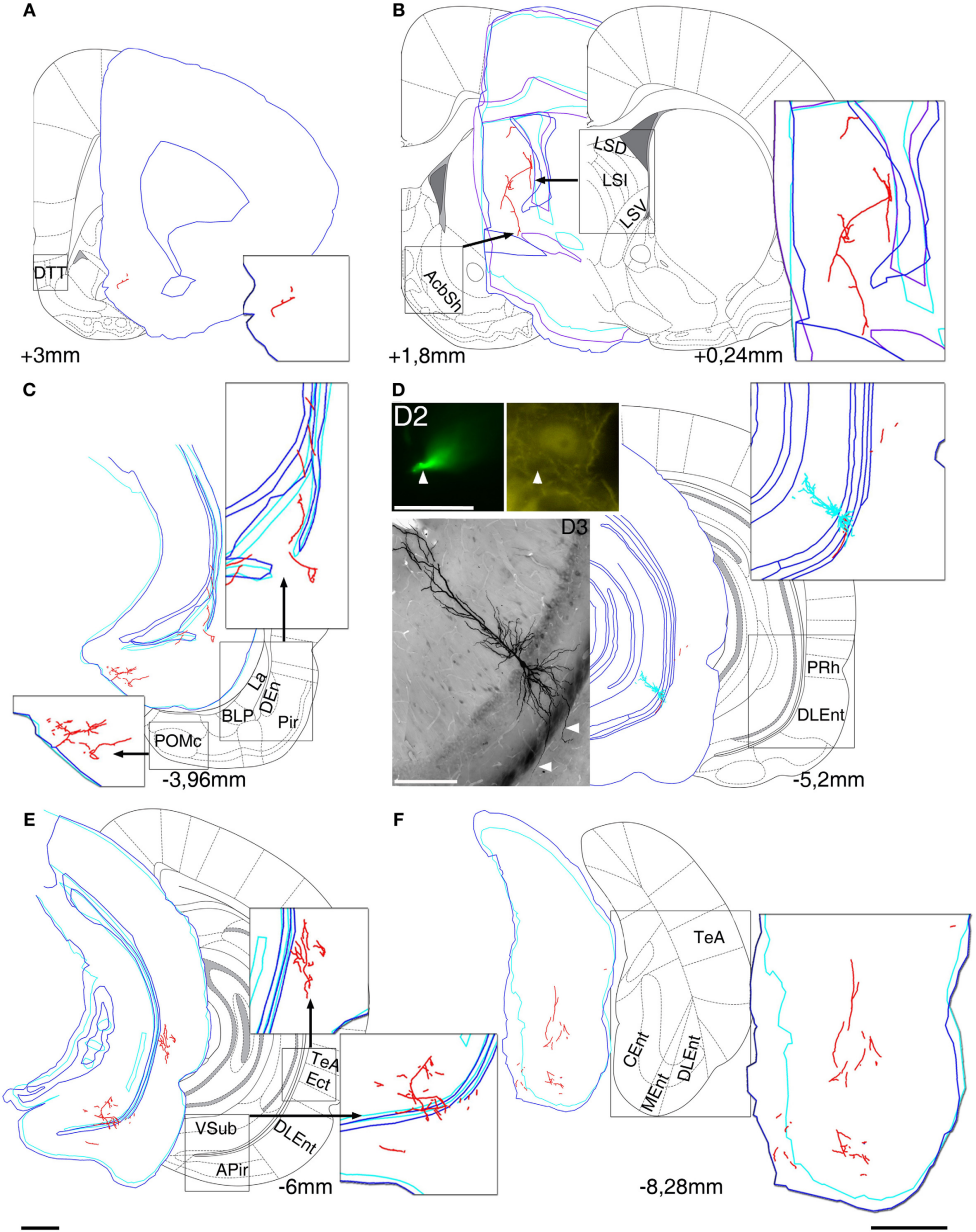
**Reconstruction of the axonal projections and somato-dendritic position of a labeled pyramidal neuron in VCA1 (A64)**. Each panel demonstrate a block of several consecutive sections (A/5 section, B/20, C/5, D/2, E/5, and F/5) reconstructed in neurolucida. Each block was correlated with the modified Paxinos atlas along the rostro-caudal axis, showing target areas where local collaterals with boutons (possible terminals) were found. A main axon (thick, myelinated) enters to the septum, and there it give rise to several preterminal axons innervating different subareas **(B)**. More anterior it bifurcates again and simultaneously innervates AcbSh **(B)** and DTT **(A)**. Different nuclei of amygdalar complex, amygdalopiriform transition area **(C)** and temporal association cortex TeA **(E)** are directly innervated. **(D)** Soma position as reconstructed (red open circle on Figure 2). Cell was calbindin (CB) negative tested on a proximal dendrite (arrowheads, **D2**). Note that the basal dendrites of the neuron perforate the alveus and enter the deep layer of lateral entorhinal cortex (arrowheads on extended focal image of 2 sections on bottom left). The caudal projection via subiculum emits local collaterals in subiculum **(E)** and terminates in different parts of the entorhinal cortex **(F)**. Drawings/atlas scale bar: 1 mm; D2/Fluorescent image scale bar 20 μm, D3/Light microscopic image scale bar: 200 μm.

To determine the axonal projections, dendritic arborizations and molecular expression of VCA1 pyramidal cells, we used the juxtacellular labeling technique and filled individual pyramidal neurons in VCA1 with neurobiotin (Figure 1). Twelve individual VCA1 pyramidal cells were analyzed further and all experiments with limited labeling, labeling of multiple cells or interneurons or cells outside the CA1 and CA2 area were excluded.

Extracellular recordings of the activity of the pyramidal cells always preceded labeling. However, most pyramidal cells in the VCA1 fired with very low rates (<0.1 Hz) in urethane anesthetized rat. In addition, VCA1 pyramidal neurons are packed in a compact layer close to each other. Therefore in most experiments the firing patterns could not be unequivocally matched with the labeled neuron and no further analysis of the firing patterns of the labeled cells is presented here.

### Position of the somata of labeled neurons

**Figure 2:**
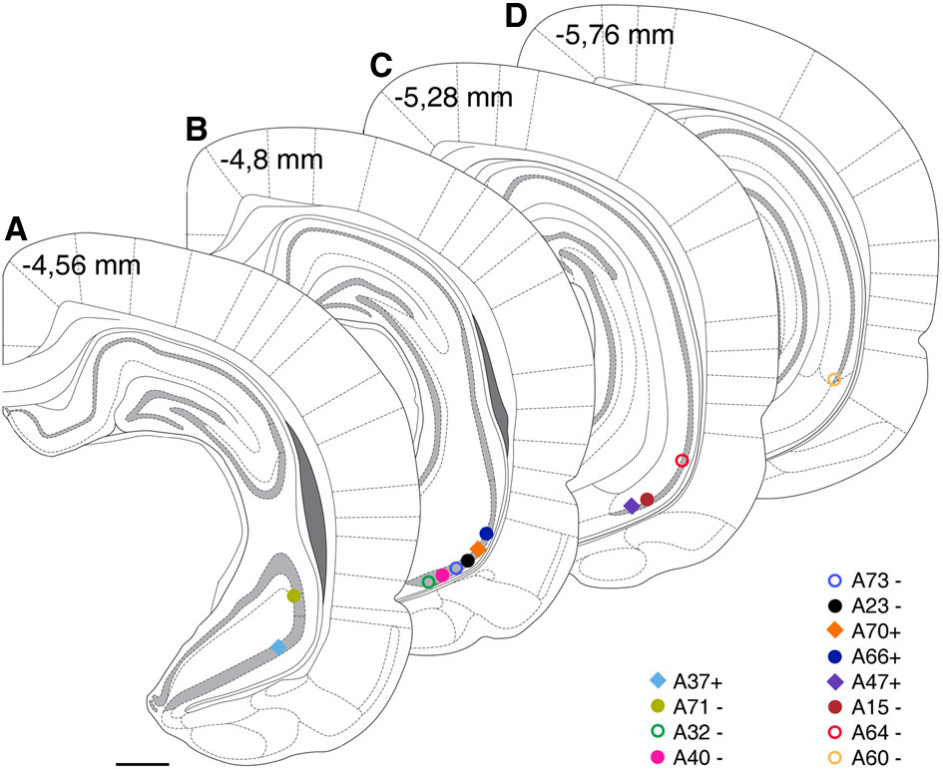
**Estimated cell body positions of neurobiotin labeled pyramidal neurons in VCA1 are projected onto coronal sections (A–D) modified from Paxinos atlas**. Individually color-coded (see later figures) shapes represents the cells included in this article: Positions of cells are represented by filled circles or filled squares, representing neurons with projection areas shown on **Figure 6**, or open circles for cells shown in **Figure 5**. Scale bar: 1 mm.

Somata of labeled pyramidal neurons were located in different positions along the antero-posterior (AP) axis (Figure 2). For most of the experiments (*n* = 10; Table 1) only a single pyramidal cell was labeled within the brain. In two cases an additional weakly labeled neuron was visible (A32, A40), but it was possible to allocate and distinguish the axonal projections during reconstruction. Two neurons were located most rostrally within stratum pyramidale of the ventral hippocampus. One cell was located in VCA1 (A37) and the other cell (A71) was located in the ventral CA2 area (Figure 2A). Most of the labeled cells (A32, A40, A73, A23, A70, A66) were located around −4.8 mm posterior to bregma (Figure 2B) and cells A47, A15, and A64 were more caudally located along the AP axis between Br −5.2 and −5.4 mm (Figure 2C). At the most caudal coordinates the VSub extends more lateral and dorsal and the CA2 gradually disappears, cells A64 and A60 were still located within the VCA1 (Figure 2D).

**Table 1:** **Molecular expression and apical dendrite structure of VCA1 pyramidal cells**.

**Cell**	**Estimated position of soma (AP)**	**Calbindin**	**Norbin**	**Single apical dendrite**	**Double apical dendrite**
A15C	−5.4	−	n.t.	+	
A23H	−4.8	−	−		+
A32C	−4.92	−	−	+	
A37C	−4.56	+	+		+
A40C	−5.04	−	+	+	
A47C	−5.3	+	+	+	
A60C	−5.7	−	+	+	
A64C	−5.2	−	−	+	
A66C	−4.8	+	n.t.	+	
A70C	−4.8	+(w)	+		+
A71S^§^	−4.56	−	+(w)	+	
A73S	−4.92	−	+	+	

C, sections cut coronal; H, horizontal; S, sagittal.

§ Cell located in CA2.

n.t., not tested; w, weak.

### Neurochemical expression profile of the labeled pyramidal neurons

**Figure 3:**
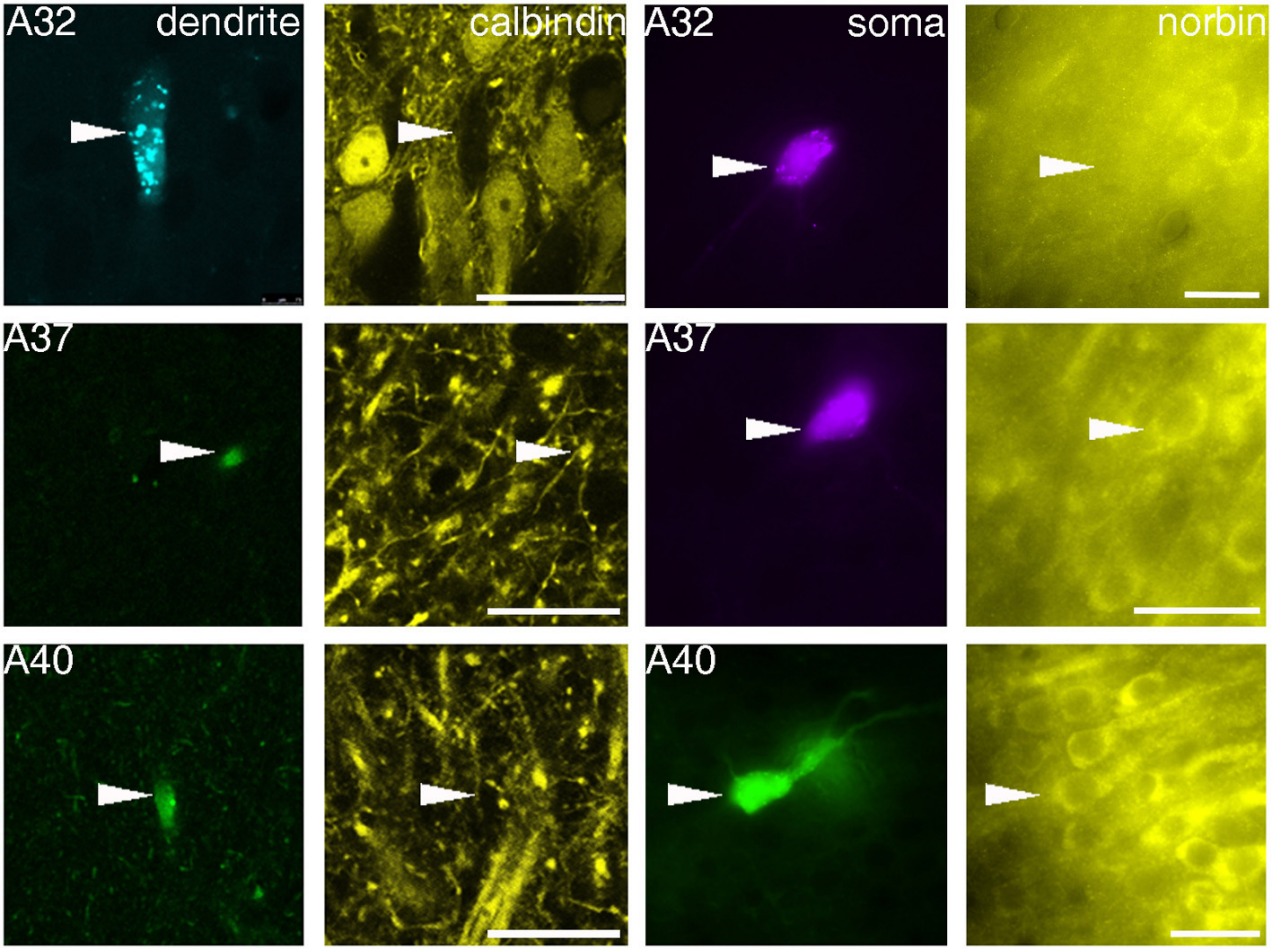
**Fluorescence micrographs showing distinct immunoreactivity of individual VCA1 pyramidal cells for calbindin and norbin**. Immunoreactivity for calbindin was tested on apical dendrites of labeled pyramidal cells (confocal images), immunoreactivity for norbin was investigated on labeled somata (Arrowheads) with an epifluorescence microscope. The three cells shown have different immunoreactivity, pyramidal cell A32 was tested as CB negative/norbin negative, cell A37 was double positive and cell A40 was tested negative for CB but positive for norbin. Scale bars: left, 25 μm; right, 20 μm.

The sections containing the soma and proximal dendrites were processed for immunohistochemistry to analyse the expression of calbindin (CB) and norbin (N) as possible markers to differentiate pyramidal neurons in VCA1. The distribution of CB immunoreactive pyramidal neurons in the dorsal CA1 were described in previous studies (Baimbridge and Miller, 1982; Rami et al., 1987; Sloviter, 1989; Celio, 1990), and we found similar expression pattern in the VCA1 i.e., the pyramidal neurons toward the stratum radiatum express detectable levels of CB. The cytosolic protein norbin (N) is localized in the somatodendritic region of most pyramidal neurons in the hippocampus (Shinozaki et al., 1999). We found that CB and N were often co-localized, however a smaller portion of CB+/N− and CB−/N+ pyramidal neurons were also found, resulting in four subgroups of the pyramidal neurons including the CB−/N− population. We tested the immunoreactivity of CB on proximal dendrites and N on the soma of neurobiotin-filled cells and found cells tested double positive, cells tested double negative and cells tested CB−/N+ (Figure 3, Tables 1, 2A). The CB+/N− phenotype was not observed amongst the filled neurons.

**Table 2A:** **Molecular expression and axonal projections of VCA1 pyramidal cells**.

**Cell**	**CB**	**N**	**1. Route**	**Target areas**	**2. Route**	**Target areas**	**3. Route**	**Target areas**
A15C	−	n.t.	+		+	APir, DLEnt	+	
A23H	−	−	n.t.		n.t.		+	DLEnt, APir
A32C	−	−	+	LSV, DTT	+	BLP, AHiPL, APir, PMCo	+	CEnt, MEnt, PaS
A37C	+	+	+	LSI	+	Pir, APir, AHiPM, DLEnt	+	AHiPM, APir, DIEnt, VIEnt
A40C	−	+	+	SFi	+	Pir, BLP, BMP, AHiPM	+	
A47C	+	+	+	LSI	+	PMCo, AHiPM, APir	+	DIEnt, VIEnt, MEnt
A60C	−	+	+	LSI, PLH	+	APir, LaVM, BLP, DLEnt	+	MEnt, DIEnt
A64C	−	−	+	LSI, LSV, LSD, AcbSh, DTT	+	BLA, LaVM/DL, CeC, BLP, BMP, PMCo, APir, Pir, DEn, TeA, PRH, Ect	+	PaS, DLEnt, DIEnt, VIEnt, MEnt, CEnt
A66C	+	n.t.	−		−		+	PrS, PaS, CEnt
A70C	+(w)	+	+		−		+	DLEnt, VIEnt,
A71S^§^	−	+(w)	+	LSI	−		+	DIEnt
A73S	−	+	+	LSI	+	BLP, La	+	DIEnt or DLEnt

C, sections cut coronal; H, horizontal; S, sagittal.

§ Cell located in CA2.

n.t., not tested; w, weak.

**Table 2B:** **Abbreviations**.

**Region**	**Abbreviation**	**Names**
Subicular complex	PrS/PaS	Presubiculum, parasubiculum
Parahippocampal region	PRh/Ect	Perirhinal cortex, ectorhinal cortex
	DLEnt/DIEnt/VIEnt	dorsolateral/dorsal intermediate/ventral intermediate entorhinal cortex (lateral entorhinal cortex)
	MEnt/CEnt	medial/caudomedial entorhinal cortex (medial entorhinal cortex)
Amygdaloid complex	BLA/BLP	Basolateral amygdaloid nucleus, anterior part/posterior part
	BMP	basomedial amygdaloid nucleus, posterior part
	LaVM/LaDL (La)	lateral amygdaloid nucleus, ventromedial part/dorsolateral part
	CeC	central amygdaloid nucleus, capsular
	STIA	bed nucleus stria terminalis, intraamygdaloid division
Amygdalopiriform transition area	APir	Amygdalopiriform transition area
	AHiPM/PL	amygdalohippocampal area, posteromedial part/posterolateral part
	PMCo/PLCo	posteromedial/posterolateral cortical amygdaloid nucleus
Septal complex	SFi	Septofimbrial nucleus
	LSD/LSI/LSV	lateral septum dorsale/intermediate/ventrale
Ventral striatum	AcbSh	Accumbens nucleus, shell
Olfactory regions	Pir	Piriform cortex
	DEn	dorsal endopiriform nucleus
	AOP	anterior olfactory nucleus, posterior part
	DTT	dorsal tenia tecta
Hypothalamus	PLH	Peduncular part of lateral hypothalamus
Association cortex	TeA	Temporal association cortex

### Pyramid cells with single or bifurcating apical dendrite

**Figure 4:**
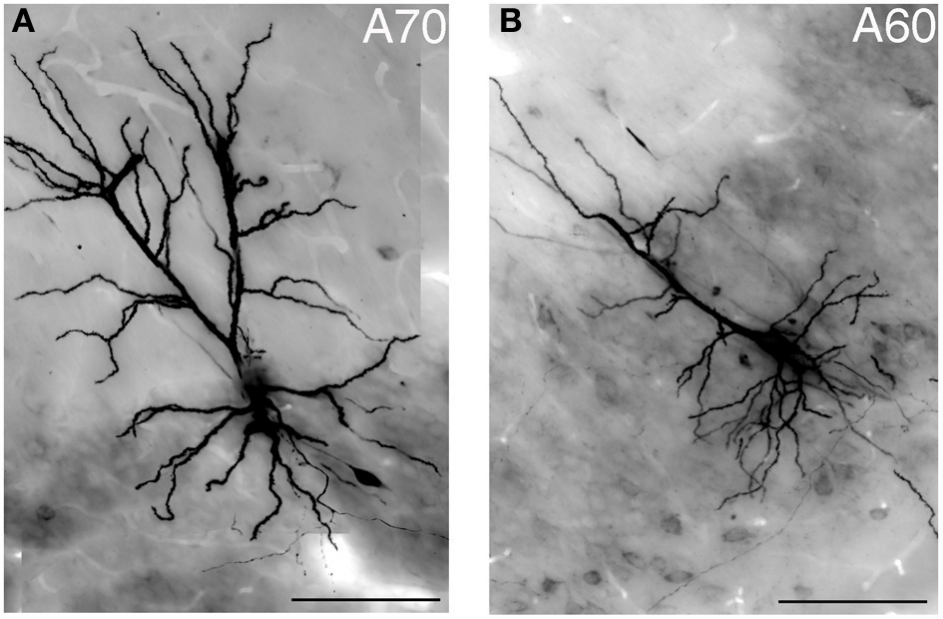
**Light micrograph showing two distinct geometries of apical dendrites for VCA1 pyramidal cells. (A)** Cell A70 has a bifurcated apical dendrite (images were taken by extended focal imaging from 3 consecutive sections with a total thickness of 210 μm), **(B)** cell A60 has a single apical dendrite (extended focal image from 1 section of 70 μm thickness). Scale bars: 50 μm.

Typically, hippocampal pyramidal cells have a single apical dendrite. However, pyramidal cells with twin-apical dendrites, bifurcating close to the soma (Bannister and Larkman, 1995; Spruston, 2008) have been described. We analyzed the dendritic geometries of the neurobiotin-filled cells (Figure 4) and observed that three out of 12 labeled pyramidal neurons had bifurcating apical dendrites, suggesting significant proportions of cells with this dendritic specification in the ventral CA1 hippocampus. However, we did not observe a correlation between dendritic arborization and molecular expression and individual pyramidal cells with bifurcating apical dendrites were tested CB−/N− or CB+/N+ or CB−/N+ (Tables 1, 2A).

In addition to differences in the apical dendrites, we found that the basal dendrites of two cells (A64, Figure 1D and a cell not shown, A75) crosses the alveus and entered into the entorhinal cortex.

### The axons of VCA1 pyramidal cells emit local collaterals and projects to distant brain areas with three different routes

**Figure 5:**
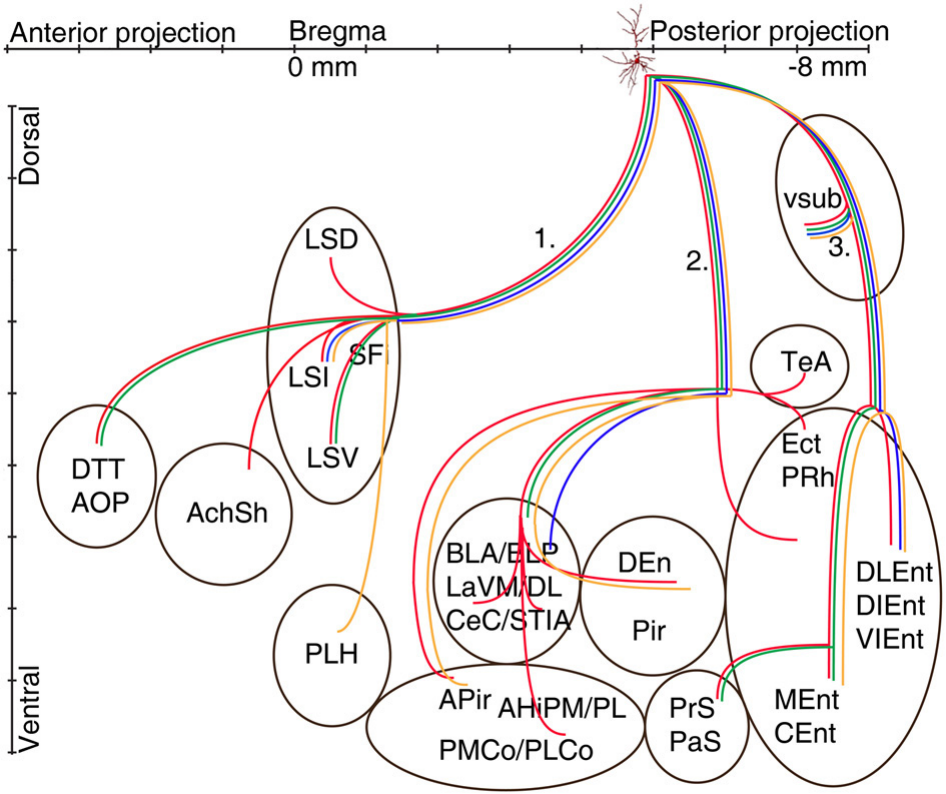
**Schematic illustration of the axonal projections and target areas of four CB-negative VCA1 pyramidal cells (A64 red, A60 orange, A32 green, A73 blue same color code as in Figure 2)**. The main axons of these cells branch in stratum oriens into three different routes: (route 1) via the fimbria/fornix system axons innervate septal nuclei and branches are reaching anterior olfactory regions (A64, A32) or (A60) turn ventrally to innervate the lateral hypothalamus. Axons of route 2 perforate the alveus and project to the amygdalar complex, amygdalohippocampal transition area or the entorhinal cortex. Branches of route 3 cross the ventral subiculum caudally from the soma to different subregions of the entorhinal cortex. (See abbreviation list).

**Figure 6:**
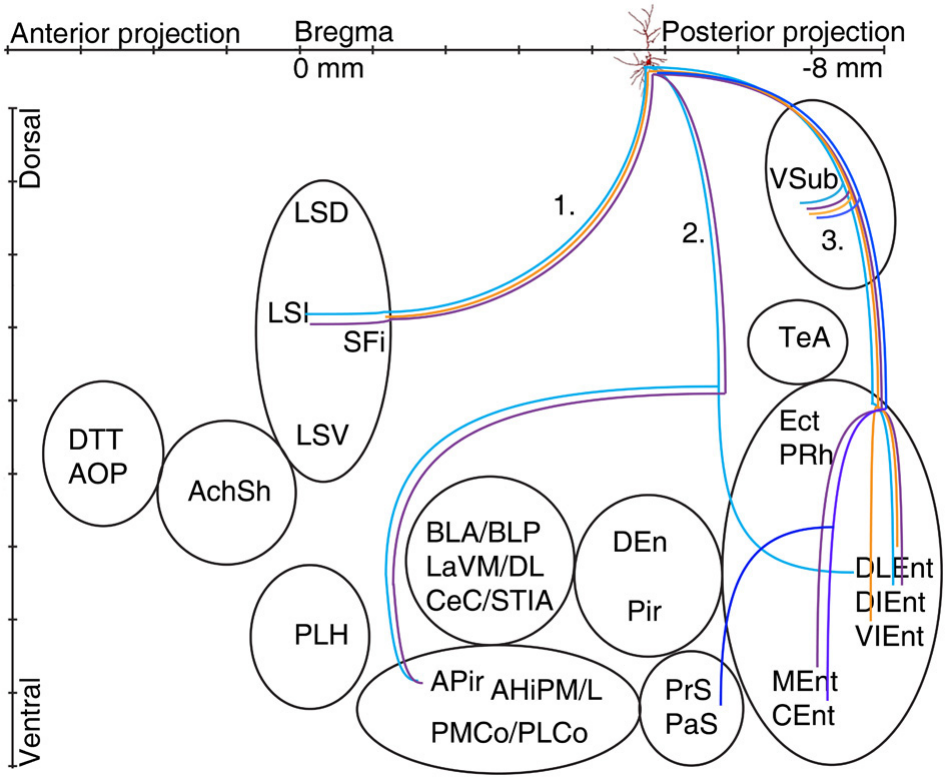
**Schematic illustration of the axonal projections and target areas for all labeled CB+ cells (A37 light blue, A47 violet, A70 orange, A66 dark blue)**. The first route reaches the septum and innervates the intermediate part (A37, A47), the second projection (not observed for cell A70 and A66) arrive to the amygdalopiriform area and cell A37 innervates entorhinal cortex (DLEnt) directly through alveus. In the third direction the cells innervate the subiculum and different parts of the entorhinal cortex.

The axons of the pyramidal neurons emerged from the basis of the soma and started to emit local branches in the stratum oriens. The main axons of all (exceptions: A70, Figure **6**, A71 located in CA2 and A66 with destroyed soma) labeled pyramidal cell divided into three major routes (Cenquizca and Swanson, 2007) to innervate their distant target areas. The first route (Figures 5, 6) ran antero-dorsally via the fimbria/fornix system and projected to septum and more anterior brain areas. The second route (Figures 5, 6), the direct pathway, avoided the ventral subiculum (VSub) and axons directly projected perpendicular through the alveus in antero-lateral direction to the amygdalar complex and/or amygdalo-hippocampal transition area and caudo-laterally to lateral entorhinal region or to olfactory related areas (Pir, DEn) or temporal association cortex (TeA). The third route ran caudally to the VSub and than innervated several lateral and medial entorhinal cortical areas (Figures 5, 6). Contralateral projections were not observed in any of the filled neurons. Often the neurobiotin-labeling of distant axonal branches faded and therefore the lack of a projection to a certain brain area cannot be concluded from these experiments.

### Local intrahippocampal axonal arborizations

Local collaterals in the stratum oriens were common in filled neurons. Some cells (Table 3) also innervated stratum pyramidale and sent few axons to radiatum. These collaterals were sparse and restricted to the close vicinity of the cell body layer.

**Table 3:** **Axonal projections within the hippocampus of VCA1 pyramidal cells**.

**Cell**	**Estimated position of soma (AP)**	**VCA1 ori**.	**VCA1 pyr**.	**VCA1 rad**.	**VSub**	**VSub mol**.	**DCA3 ori**.	**DCA3 pyr**.
A15C	−5.4	+++			++			
A23H	−4.8	+++		++	++++			
A32C	−4.92	++			+			
A37C	−4.56	++			+++	++		
A40C	−5.04	++			+			
A47C	−5.3	+++	++	+	+++	++		
A60C	−5.7	++	++	++	+++			
A64C	−5.2	++			+++			
A66C	−4.8	++	+		+++	+++		
A70C	−4.8	+++	++	++	++++	++		
A71S^§^	−4.56	+++	++	+++	++++		++	++
A73S	−4.92	++	+		+++			

C, sections cut coronal; H, horizontal; S, sagittal.

§ Cell located in CA2.

All of the neurons were innervating VSub pyramidal layer but the density of local collaterals and boutons differed amongst individual cells (Table 3). The molecular layer of the VSub was exclusively innervated by all the four CB+ cells. Cell A71, located in VCA2, sent few collaterals to dorsal CA3 stratum oriens and stratum pyramidale (Table 3).

### Extrahippocampal projections of CB negative pyramidal neurons

Eight out of 12 labeled neurons (Tables 1, 2A,B) were tested negative for CB. These cells had dense projections via all three major routes of axonal targets.

The first route of CB− cells runs antero-dorsally to the lateral septum, where axons of each cells innervated one of the subregions of the lateral septum intermediate part (LSI), ventral part (LSV), septofimbrial nucleus (SFi). As an exception, the axon of A64 was immediately branching as it entered the septal area and innervated the dorsal part (LSD) as well as the LSV and the intermediate segment (LSI) (Figures 1B, 5) The axons of two cells (A32, A64) could be followed more rostrally where they innervated the dorsal tenia tecta (DTT) (Figures 1A, 5) and the anterior olfactory nucleus posterior (AOP). One axonal branch of cell A64 reached the nucleus accumbens shell (AcbSh) (Figures 1B, 5). Another cell (A60) was found to project caudo-ventrally to the peduncular part of the lateral hypothalamus (PLH) through the septum (Figure 5).

The second main route ran antero-laterally to the amygdalar complex (BLA, BLP, BMP, LaVM/DL, CeC and STIA) where the axon of cell A73 ended in the BLP and in lateral amygdaloid nucleus (LaVM/DL). Other cells (Figure 5, A32, A60, A64) entered into the amygdala and branched there (Tables 2A,B, Figure 5). Branches of A64 innervated many amygdalar nuclei (BLA, BLP, LaVM/DL, CeC, and STIA). The main axon of A60, A64 entered to piriform areas (Pir, DEn), and an axon bifurcated before the amygdala and, avoiding the amygdala, ran antero-laterally to the amygdalo-piriform transition area (APir). Axon collaterals of A64 and A32 innervated the amygdalohippocampal transition area (PMCo, PLCo). Furthermore cell A64 innervated caudo-laterally the temporal association area (TeA), entorhinal (DLEnt), peri- and ectorhinal regions (PrH, Ect).The third route of axonal projections ran caudally to the subiculum, sent local collaterals to subicular neurons more caudally and ended in the medial entorhinal cortex (MEnt, CEnt) and/or in the lateral entorhinal cortex (DLEnt, DIEnt, VIEnt). Cells A64 and A32 also projected to pre- and parasubiculum (PrS, PaS).

### Extrahippocampal projections of CB positive pyramidal neurons

The axon of CB+ pyramidal cells (A37, A47, A66, and A70; Tables 2A,B, Figure 6) were innervating the LSI via route 1, but the fading axon did not allow to follow their possible route to more anterior brain areas. The second (direct) innervations ran anterior (A37, A47) to amygdalo-hippocampal target regions, the amygdaloid complex seemed to be avoided by these cells. Collaterals from cell A70 to the 2nd route were not found. This cell, in addition the faint route 1 branch, innervated strongly the VSub on the route 3 and only one axon projected further to the medial (DLEnt) and caudolateral (VIEnt) entorhinal cortex. Cell A37 had collaterals to DLEnt from the direct route 2 to DIEnt, VIEnt by VSub. The neuron A47 innervated entorhinal areas (DIEnt, VIEnt and MEnt) via the third route. Cell A66 had no antero-dorsal (via 1.) or antero-lateral (via 2.) projection but caudally (via 3.) projected to PrS, PaS and finally entered to the medial entorhinal cortex (CEnt).

## Discussion

We have used the juxtacellular labeling method (Pinault, 1996) to visualize individual pyramidal neurons in the ventral hippocampus and observed distinct molecular expression of calbindin and norbin, different arborizations of the apical dendrites and that pyramidal cells of the ventral hippocampus send long-range projections via three major and distinct routes to different brain areas.

### Dendritic arborizations

Lorente De No (1934) reported pyramidal neurons with twin apical dendrites. We found three out of 12 examined pyramidal cells with such bifurcating apical dendrites. All of them were located close to the border between strata pyramidale and radiatum, similarly to earlier studies in ventral subiculum (Harris et al., 2001). A bifurcating apical dendrite may provide distinct integration of input signals but it has not been studied in detail. So far no correlations of bifurcating apical dendrites with intrinsically bursting or regular spiking patterns have been observed.

For two cells we observed dendrites crossing the white matter between the VH and the entorhinal cortex (A64 in Figure 1D and a cell not shown, A75). Although the synaptic inputs to these dendrites in the entorhinal cortex are unknown, a dendritic integration of inputs from a different brain area may provide additional complex integration possibilities for hippocampo-entorhinal circuit operations.

The dendritic arborization were also examined but we were unable to find any group forming differences in our sample as it was shown earlier in slice (Graves et al., 2012).

### Axonal projections and routes

The main axon of a CA1 pyramidal neuron leaving the soma emits preterminal axons forming boutons with synapses onto target dendrites, then bifurcates into several myelinated main axons forming the routes toward different brain areas. To understand how the information of firing patterns is distributed to different target areas it seems important to map not just the terminal fields but also the branching pattern of the projection axons.

Tracers used by bulk injections are the most often used tools to map projections in the brain providing accurate data on the terminal field distribution. On the other hand when it comes to the mapping of routes it may have the disadvantage of problematic differentiation of crossing but not bifurcating fibers in a field of dense axonal labeling. Nevertheless using bulk labeling Cenquizca and Swanson (2007) successfully differentiated different routes for the projecting axons of ventral CA1 pyramidal cells, a ventral projection through the longitudinal association bundle passing through the VSub first, and a direct rostral projection involving the fornix.

Our results suggest that the main axon of individual VCA1 pyramidal neurons branches in stratum oriens close to the soma and projects into three different routes. The first route travels anterio-dorsally through the fimbria/fornix system (ffxs), entering the white matter in the alveus and running toward septum and even more anterior brain areas, but avoiding VSub. The second axonal route is a direct pathway, and the main axonal branch of this route leaves the VH by crossing the alveus straight toward the EC and innervating several brain regions. The existence of this route was proposed earlier (Cenquizca and Swanson, 2007) and is confirmed here. The axons via the third and classical route enter the VSub forming terminal fields and main axons caudally exit to the EC. Contralateral projections were not observed in the labeled pyramidal cells, confirming previous results by Witter and Amaral (2004), that CA1 principal cells do not have extensive commissural projections. Our results show that most pyramidal cells of the ventral CA1 hippocampus send axonal projections via each of these three main routes.

### Terminal fields of VCA1 pyramidal neurons

All the three mentioned routes are responsible for delivering the axons to separate sets of brain areas. The first pathway, via the ffxs, enters the septal area innervating it and crossing to more anterior areas described earlier (Swanson and Cowan, 1977; Risold and Swanson, 1996). A topographic organization of mainly the dorsal hippocampal projections to the lateral septum has been described (Tamamaki et al., 1987; Amaral and Witter, 1989; Amaral et al., 1991; Tamamaki and Nojyo, 1991; Zhou, 1997), axons originating from the medial hippocampus innervates the dorsal part, whereas axons from the lateral hippocampus reach the ventral part of LS. Most of our cells innervated only one region of LS, supporting the existence of a topographical projection from the VH. However, for one labeled pyramidal cell A64 we found that the axon was innervating all lateral septal subnuclei. Additional brain areas have been described to receive hippocampal input via this route (Cenquizca and Swanson, 2006, 2007), but we were not able to follow filled axons to the infralimbic-, orbital-, prelimbic- and anterior cingulate cortex or in ventral tenia tecta (VTT), most likely because of the incomplete filling of the axons.

The second axonal route, directly crossing the alveus and avoiding VSub, targeted the amygdalar complex (BLA, BLP, LaVM,/DL, CeC), bed nucleus stria terminalis interamygdaloid division (STIA), traveled and innervated also amygdalo-piriform transition area (APir, AHiPM/L, PMCo/PLCo), piriform area (DEn, Pir), ecto-, peri and entorhinal area. Cell A64 exceptionally innervated TeA, Ect, and PRh, while cell A37 projected to DLEnt which is usually innervated via the 3rd route through the VSub.

The best known 3rd projection route passed through the VSub and innervated the medial part (MEnt, CEnt) and the lateral part (DLEnt, DIEnt, VIEnt) of the entorhinal cortex as it was described earlier (Cenquizca and Swanson, 2007). Although the three major projection routes were used by most pyramidal cells, it is likely that the terminal field and synaptic innervations might differ between individual pyramidal cells.

Pyramidal neurons were suggested to participate in two separate circuits in the VH one controlling the neuroendocrine activities, connecting VCA1 to VSub, BLP, LSV, and BST and the second circuitry may play role in fear learning via direct connections from VH to the CeC (Canteras and Swanson, 1992; Risold and Swanson, 1996; Cenquizca and Swanson, 2007). In contrary we found a cell (A64) projecting to both the CeC and to the LSV, VSub, BLP, and BST (STIA), combining the two circuitry while the cell A32 innervated the LSV, VSub, BLP but avoided CeC fitting into the hypothesis (Tables 2A,B). All the rest avoided CeC but also LSV and rather terminated in LSI. This indicates that even if the two suggested directions exist there could be a third group combining them.

All pyramidal cells had local collaterals in VCA1 in neighboring str oriens and few in the pyramidale and radiatum. The pyramidal layer of VSub was innervated by all cells but only CB+ neurons innervated the molecular part of VSub.

Overall, our data suggest that all types of information encoded by pyramidal cells in VCA1 are conveyed to other parts of the brain via all three major VCA1 projection routes. However, differences in the terminal fields of these projection routes might exist between distinct VCA1 pyramidal cells and it is possible that these differences correlate with the molecular expression or dendritic branching of different pyramidal cells.

### Molecular expression, neurochemical profile

We used antibodies against norbin and calbindin to identify neurochemical subgroups among VH pyramidal cells. CB+ cells are found in the compact layer of the str. pyramidale whereas CB− cells preferentially found in the part of the pyramidal layer that is closer to the str. oriens. Subsets of the neurobiotin-labeled pyramidal cells were expressing different combinations of these two proteins. We did not observe a correlation of Norbin expression with axonal and dendritic arborizations, however a difference in the local innervation (VSub mol.) and in the amount of long range projections was observed between CB+ and CB− neurons in spite of similar juxtacellular labeling quality and time (CB−: average time between cell labeling and perfusion was 16 h 37 min and the average modulation length were 343 s, for CB+ the average time between cell labeling and perfusion was 16 h 5 min, the average modulation was 400 s). The older CB negative cells had more widespread and dense innervation patterns throughout the brain compared to the later developing CB+ cells (Baimbridge et al., 1991), and this difference may also correlate with the difference in carrying spatial (CB−) or non-spatial (CB+) information (Mizuseki et al., 2011). However, because of the incomplete filling of the cells reported here further verifications will be required to confirm this observation.

### Outlook

It has been demonstrated that the ventral pole of the hippocampus is involved in anxiety and stress responses (Moser and Moser, 1998; Bannerman et al., 1999, 2002, 2004), supported by widespread efferent and afferent connections. The differences among VCA1 pyramidal neurons in their neurochemical profile or extension of their projections shown here adds to the complexity of this area. We have shown here that most VCA1 pyramidal cells send projection axons via the three main routes. It remains to be investigated if the terminal fields of the three major projection routes differ between distinct pyramidal cells and which information is transferred via these axonal fibers.

### Conflict of interest statement

The authors declare that the research was conducted in the absence of any commercial or financial relationships that could be construed as a potential conflict of interest.

## Abbreviations

AOP: anterior olphactory nucleus, posterior part
DTT: dorsal tenia tecta
AcbSh: accumbens nucleus, shell
ffxs: fimbria/fornix system
LSD: lateral septum dorsale
LSI: lateral septum intermediate
LSV: lateral septum ventrale
SFi: septofimbrial nucleus
PLH: peduncular part of lateral hypothalamus
BLA: basolateral amygdaloid nucleus, anterior part
BLP: basolateral amygdaloid nucleus, posterior part
CeC: central amygdaloid nucleus, capsular part
LaVM: lateral amygdaloid nucleus, ventromedial part
LaDL: lateral amygdaloid nucleus, dorsolateral part
LaVL: lateral amygdaloid nucleus, ventrolateral part
STIA: bed nucleus stria terminalis, intraamygdaloid division
APir: amygdalopiriform transition area
AHiPM: amygdalohippocampal area, posteromedial part
AHiPL: amygdalohippocampal area, posterolateral part
PMCo: posteromedial cortical amygdaloid nucleus
PLCo: posterolateral cortical amygdaloid nucleus
Pir: piriform cortex
DEn: dorsal endopiriform nucleus
PrS: presubiculum
PaS: parasubiculum
PRh: perirhinal cortex
Ect: ectorhinal cortex
DLEnt: dorsolateral entorhinal cortex
DIEnt: dorsal intermediate entorhinal cortex
VIEnt: ventral intermediate entorhinal cortex
MEnt: medial entorhinal cortex
CEnt: caudomedial entorhinal cortex
TeA: temporal association cortex
DH: dorsal hippocampus
VH: ventral hippocampus
VSub: ventral subiculum.
